# Technical Complications and Marginal Bone Loss Depending on the Crown Material of Dental Implants in the Posterior Region: A 3-Year Randomized Clinical Study

**DOI:** 10.3390/dj13090430

**Published:** 2025-09-17

**Authors:** Sigmar Schnutenhaus, Marla Weinmann, Max Römer, Ralph G. Luthardt

**Affiliations:** 1 Center for Dentistry, Dr. Schnutenhaus Community Health Center (CHC) GmbH, Breiter Wasmen 10, 78247 Hilzingen, Germany; 2 Department of Prosthetic Dentistry, Center of Dentistry, Ulm University, Albert-Einstein-Allee 11, 89081 Ulm, Germany; 3 Privat Practice Zahnmedizin Römer, Windacher Höhe 2, 86949 Windach, Germany

**Keywords:** dental implant, success rate, technical complications

## Abstract

**Background/Objectives:** This single-center, randomized controlled clinical trial evaluated the impact of two crown materials—lithium disilicate (LS2) and a polymer-infiltrated hybrid ceramic (HC)—on the marginal bone loss (MBL) and the technical complications in implant-supported single-tooth restorations over a three-year period. **Methods:** Sixty patients with posterior single-tooth gaps were randomly assigned to receive either LS2 or HC crowns on iSy (Camlog) implants. All of the restorations were fabricated as CAD/CAM-based hybrid abutment crowns bonded to prefabricated titanium bases. Standardized radiographs were taken at the baseline (T0) and at three years (T1) to assess the MBL using ImageJ software. The technical complications were prospectively recorded. The data analysis was descriptive and exploratory. **Results:** Fifty-eight cases were available for the final evaluation. The three-year implant survival rate was 100%. The mean marginal bone remodeling was minimal (mesial: LS2 0.15 mm, HC 0.08 mm; distal: LS2 0.13 mm, HC 0.12 mm), with no statistically significant intergroup differences. Bone apposition was observed in 74.1% of the cases. The male patients showed a significantly greater mesial bone loss (*p* = 0.024). Technical complications occurred more frequently in the HC group, including crown fractures (25%), decementation (17.9%), and screw loosening (14.3%). In the LS2 group, only screw loosening (12.5%) was observed. **Conclusions:** The lithium disilicate-based hybrid abutment crowns demonstrated a high clinical reliability with stable peri-implant bone and fewer technical complications over three years. In contrast, the hybrid ceramic crowns were associated with a higher rate of mechanical failure. Material selection should therefore be a key consideration in planning implant-supported single-tooth restorations.

## 1. Introduction

The success of implant-supported single-tooth restorations is often assessed based on the survival rate of the implants, as this allows conclusions to be drawn about the risk of implant loss. However, a consideration of osseointegration alone is insufficient, as the long-term success of a restoration is also defined by the functional stability, the freedom from complications, and the long-term integrity of the prosthetic components. Against this background, the present study is dedicated to analyzing the clinical success of implants and their prosthetic restoration in the posterior region over a period of three years. Meta-analyses show that the five-year survival rates for implants supporting single crowns range between 94.5% and 97.2%, depending on the crown material and the study design, though even these high success rates are accompanied by significant biological, technical, and aesthetic complication rates [1,2,3].

The Pisa Consensus Conference (2007) definition is often used to assess implant success. In addition to clinical parameters such as freedom from pain, stability, and freedom from exudate, this definition specifies radiographically detectable bone loss of less than 2 mm as a decisive criterion [4]. Marginal bone loss (MBL) is subject to a variety of individual influencing factors. In addition to non-modifiable risk factors such as genetic preposition or anatomical conditions, behavioral factors, in particular tobacco consumption, are among the most significant influencing factors. A meta-analysis has shown that the MBL is lowest in non-smokers and increases significantly with increasing cigarette consumption [5]. Another relevant risk factor is existing or previous periodontitis. Significantly higher bone loss rates are observed, particularly when rough implant surfaces are used [6].

On the other hand, there are protective factors that can reduce the risk of marginal bone loss. These include regular professional teeth cleaning, which significantly reduces the risk of peri-implant diseases [7]. Avoiding repeated abutment changes has also proven to be beneficial. A single-stage placement of the final abutment is associated with a lower MBL rate [8]. Bruxism, on the other hand, is a significant risk factor for the long-term stability of dental implants and for technical complications in prosthetic restorations [9].

The type of prosthetic restoration also has a significant influence on the long-term outcome. In particular, the type of fixation (cemented vs. screw-retained) and the design of the crown play a central role in terms of the biological and the technical stability. While both types of restorations carry potential risks, peri-implantitis is considered the most serious biological complication that can occur with both cemented and screw-retained reconstructions [10,11]. However, screw-retained restorations tend to show more favorable biological parameters, such as lower bleeding tendency (BOP) and reduced plaque accumulation, which are associated with a lower risk of inflammation [12,13]. A decisive disadvantage of cemented constructions is the risk of cement retention in subgingival areas. Remaining cement residues can trigger inflammatory reactions, biofilm formation, and subsequent bone loss [14].

Hybrid abutment crowns offer a promising alternative in this context. These constructions consist of a titanium base onto which the ceramic superstructure is cemented extraorally so that the excess cement can be removed under visual control. The finished crown is then screwed into place. A one-year survival rate of 98.15% is reported for this restoration concept [15]. The crown material used also has a significant influence on the long-term prognosis. Zirconium dioxide impresses with its high fracture resistance and, in combination with titanium bases, shows an improved mechanical performance compared to single-piece zirconium abutments [16]. Lithium disilicate is also considered a suitable material due to its aesthetic properties, translucency, and good machinability in CAD/CAM processes [17]. Hybrid ceramics (PICN) combine the hardness of ceramics with the elasticity of polymer components and are characterized by advantageous mechanical properties, particularly with regard to fracture behavior and marginal fit [18].

The elastic modulus of the implant and the restorative materials is a key determinant of the stress distribution at the implant-bone interface. Lower-modulus materials may reduce the peak stresses in the cortical bone, while stiffer materials can increase the load concentration [19,20,21]. Recent reviews emphasize that adjusting the elastic modulus of the biomaterials could improve their long-term outcomes [22]. This highlights the clinical relevance of evaluating its influence on implant-supported restorations.

The increasing digitalization of prosthetic workflows has contributed to the establishment of efficient treatment strategies. Intraoral scans (IOS) in combination with CAD/CAM-fabricated restorations enable a significant reduction in the treatment time for single-tooth restorations [23]. Despite the overall high success rates, the incidence of technical complications remains a clinical challenge. Late implant losses are often due to a prosthetic or functional overload [24]. A distinction must be made between biological and technical complications. The latter affect the mechanical components of the system, including the implant, abutment, screw connection, and superstructure. The most serious technical complication is implant fracture, which occurs in about 0.14% of single implants [25], mostly due to chronic overload. Screw loosening occurs more frequently, with an incidence of 8.8% after five years [1], and is influenced by factors such as the type of fixation, the implant-abutment design, and the axial deviations [26,27].

The loss of crown retention is the second most common technical complication, at 4.1% [1]. Metal-ceramic crowns that have been conventionally cemented have a higher failure rate (5.5%) than adhesively bonded ceramic crowns, where the rate is only 1.1% [28]. Here, too, the influence of the choice of the material on the stability of the restoration is evident. Another frequently observed problem is chipping, especially in the posterior region. With an incidence of 3.5% after five years [1], it impairs both the function and the aesthetics of the restoration. Due to the lack of proprioceptive feedback, implants have up to 8.7 times less tactile sensitivity than natural teeth, which can lead to an increased stress on the prosthetic components [29].

Overall, the correlations presented clearly show that the long-term success of implant-supported single tooth restorations is significantly influenced by the targeted selection of suitable materials and precise prosthetic planning. Both biological and technical complications are closely linked to the restoration design, material properties, and patient-specific risk factors and should therefore be considered in an integrated manner.

This clinical study investigates whether the crown material used, particularly in terms of its modulus of elasticity (E-modulus), has an influence on the biological and technical success of implant-supported single tooth restorations. This analysis focuses on a comparison between lithium disilicate ceramics (LS2s) and hybrid ceramic materials (HCs) with a polymer component, which differ significantly in terms of their mechanical properties. The hypothesis is tested that crowns made of HC lead to a reduced stress transfer to the implant and the peri-implant tissue due to their lower E-modulus and the associated damping of the material properties. This reduced stress concentration could manifest clinically in lower marginal bone loss and a lower incidence of technical complications compared to LS2 crowns. The aim of this study is to systematically record the potential differences in the medium-term performance of both materials over a period of three years and to evaluate their clinical relevance.

## 2. Materials and Methods

### 2.1. Trial Design

The present study is a single-center, randomized controlled clinical trial with blinded data analysis. This design was chosen to ensure standardized treatment protocols, consistent follow-up procedures, and a homogeneous patient cohort, thereby strengthening internal validity. Nevertheless, it is acknowledged that the single-center design may limit external validity and the generalizability of the results. The aim was to evaluate the biological and technical success of two different crown materials in implant-supported single-tooth restorations in the posterior region over a period of three years. A total of 60 patients who required implant-supported prosthetic restoration of a single tooth in the maxillary or mandibular arch were included in this study.

### 2.2. Ethical Considerations

This study was approved by the Ethics Committee of the University of Ulm under file number 327/15 (approval date: 29 October 2015). This prospective study is registered in the German Clinical Trials Registry (DRKS) under DRKS ID: DRKS00009628 (registration date: 9 November 2015).

All participants were fully informed about the study objectives, procedures, and possible risks prior to treatment. A written consent form was obtained from each patient at the start of this study.

### 2.3. Sample Size, Randomization, and Blinding

As there was insufficient prior clinical data available for the primary endpoint, no formal case number estimate could be made. Consequently, this study was designed in an exploratory manner. These findings should therefore be interpreted with caution and considered as a basis for the planning of future multicenter trials with adequate statistical power. The case number of 60 patients (30 per study group) is based on experience from comparable studies in which this group size is considered methodologically appropriate.

The study participants were randomized using a concealed randomization procedure with neutral, opaque envelopes. A total of 60 envelopes were prepared in advance by an independent person not involved in this study and numbered consecutively in a 1:1 ratio (lithium disilicate ceramic vs. hybrid ceramic). The envelopes were kept secure until use. Assignment to the respective study group was performed on an individual patient basis immediately after implant placement. A member of the study staff randomly selected an envelope, opened it, and documented the group assignment. This procedure ensures concealed randomization, prevents systematic bias (selection bias), and ensures an even distribution of participants between the two intervention groups.

The evaluation was conducted in a single-blind manner: while the treating clinicians were aware of the allocation, the outcome assessment and the statistical analyses were performed by independent evaluators who were blinded to group assignment. The blinding was lifted only after completion of data collection and statistical evaluation. Due to the limited sample size and the low number of technical complication events, formal subgroup analyses were not feasible. For the variable material, complication rates were summarized descriptively. In addition, a Kaplan-Meier survival curve was generated for HC crowns to visualize failure-free survival. No log-rank testing or Cox regression was performed, as the event numbers were insufficient for reliable inference.

#### 2.3.1. Inclusion Criteria

Only patients who met all of the following inclusion criteria were considered for participation in this study:

- Presence of a single tooth gap in the upper or lower posterior region with indication for implant-supported prosthetic restoration;
- The tooth loss occurred at least three months ago (late or early implantation);
- The gap to be filled was bordered by adjacent natural teeth or implants;
- There was a natural tooth on the contralateral side for functional reference;
- Antagonistic dentition was completely present;
- Written informed consent to participate in this study was obtained.

#### 2.3.2. Exclusion Criteria

Patients with one or more of the following characteristics were excluded from participation in this study:

- Age under 18 or lack of legal capacity;
- Presence of untreated periodontal disease with staging > stage II and/or grading B or C;
- Heavy nicotine use (more than 10 cigarettes per day);
- Taking bisphosphonates;
- Pregnancy;
- Alcohol or drug addiction;
- Diagnosed infectious diseases such as hepatitis B/C or HIV/AIDS;
- Uncontrolled or severe diabetes mellitus;
- Patients who wear mouth guards due to severe bruxism;
- Need for immediate implantation;
- Primarily recognizable extensive augmentation requirements, such as a sinus lift.

### 2.4. Clinical Procedure and Intervention Groups

After preliminary examination and consultation, suitable patients were included in this study. All surgical and prosthetic procedures were performed in a standardized manner by an experienced practitioner (SiS). For preoperative planning of the implant position and to assess any need for augmentation, a digital volume tomography (CBCT) with a resolution of 0.2 mm voxel was performed on all patients at the start of this study (Gendex CB500, Gendex Dental Systems, Des Plaines, IL, USA).

The implant position was determined virtually using the CBCT data and SMOP planning software (SMOP 2.6.; Swissmeda, Zurich, Switzerland) in accordance with prosthetic specifications. The planning was documented in at least sagittal and lateral views and archived using screenshots. In cases where the facial cortical bone thickness was <2 mm, simultaneous bone augmentation was performed. For this purpose, autologous bone was combined with a xenogeneic bone substitute material and covered with a resorbable collagen membrane.

The iSy screw implant (ALTATEC GmbH, Wimsheim, Deutschland) was used as the implant system. The implants were inserted according to the manufacturer’s protocol and left to heal openly. The wound was closed without tension using a single-single-button suture and monofilament polyamide suture material (Resolon 5/0, Resorba, Nuremberg, Germany). The sutures were routinely removed seven days after surgery.

The impression for the prosthetic restoration was taken depending on the bone consistency after a healing period of eight weeks for compact bone and twelve weeks for spongy bone structure or in cases of augmentation accompanying implantation. A fast-setting vinyl polysiloxane impression material (Imprint 4 Super Quick, 3M Espe, Seefeld, Germany) was used. The impression was taken using the closed tray technique with the iSy multifunctional cap. Individual trays were fabricated from Erkoplast PLA-W material (Erkodent Erich Kopp GmbH, Pfalzgrafenweiler, Germany).

After removal, the impression tray was prepared for digitization with an implant duplicate (identical diameter to the original implant). The impression was then scanned and archived as an STL data set. The corresponding master model was fabricated using a super-hard plaster (HS-CAD/CAM plaster, Henry Schein Inc., Melville, NY, USA) specially designed for CAD/CAM processing.

### 2.5. Hybrid Abutment Crowns

Two different materials were used in this study to fabricate the one-piece, screw-retained hybrid abutment crowns:

- Lithium disilicate ceramic (IPS e.max CAD, Ivoclar Vivadent AG, Schaan, Liechtenstein);
- Hybrid ceramic with polymer content (VITA Enamic, VITA Zahnfabrik GmbH, Bad Säckingen, Germany).

The materials were allocated randomly. Both crown types were cemented extraorally onto a standardized titanium base, and any cement residue was removed. The finished hybrid abutment crowns were then screwed in place using the torque recommended by the manufacturer (20 Ncm). The screw channel was closed with Teflon tape and sealed with composite (Ceram-X mono, Dentsply DeTrey, Konstanz, Germany).

All dental work was carried out centrally by dental technicians from the same dental laboratory to ensure consistent quality and standardized production. Production was carried out in accordance with the manufacturer’s protocol. Particular attention was paid to a consistent surface treatment process for the crowns. The manufacturer’s specifications for the firing process and polishing protocol were strictly adhered to.

### 2.6. Radiological Analysis of Marginal Bone Loss

To assess marginal bone loss, standardized digital intraoral single-tooth X-rays were taken at two defined time points:

- T0: 1 week after insertion of the superstructure (baseline);
- T1: 3 years after implantation.

For reproducible repositioning of the X-ray film holders, individual positioning templates made of transparent plastic material were fabricated on plaster models in the dental laboratory.

**Figure 1:**
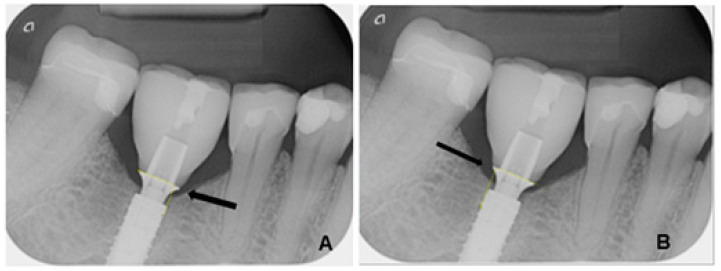
Measurement of marginal bone height on an implant at the site of a premolar mesially (**A**) and distally (**B**) between the titanium adhesive base and the alveolar ridge (yellow lines).

The radiological images were evaluated using the image analysis software ImageJ (Image Processing and Analysis in Java, version 1.53, macOS Monterey 12.7.4), a scientifically established open-source software for quantitative image processing. The measurements were calibrated based on the known diameter of each implant used. The measurement of marginal bone loss was performed both mesially and distally, orthogonally to the implant shoulder. The measurement was taken from the outer edge of the implant shoulder to the crestal bone edge (Figure 1). The examiners were calibrated prior to the measurement. In addition, several series of repeat measurements were performed at different times to reduce intraobserver variations.

All X-ray images were zoomed in for measurement to ensure maximum precision. Two bone measurements and one control measurement of the implant diameter were performed per patient at each measurement time point. The results were numbered consecutively, systematically documented, and archived in an Excel spreadsheet. Marginal bone loss was determined by calculating the difference between the baseline value (T0) and the follow-up measurement (T1).

### 2.7. Recording Technical Complications

**Figure 2:**
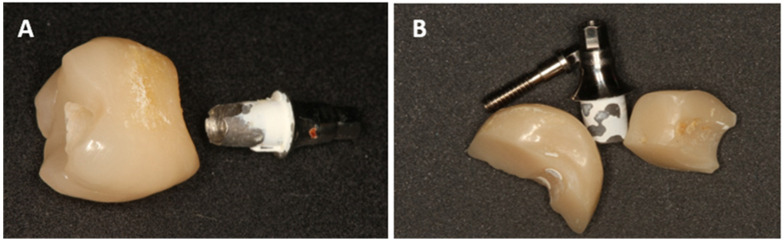
Decementation (**A**) and fracture of the hybrid abutment crown (**B**).

In addition, all technical complications were systematically recorded and documented during the observation period. These included in particular:

- Chipping or fractures of the all-ceramic superstructures (including photo documentation);
- Damage to the implant-abutment complex (Figure 2);
- Loosening or fractures of abutment screws.

Documentation was carried out continuously during the check-ups and was evaluated in terms of quality and quantity.

### 2.8. Statistical Evaluation

The statistical analysis was performed using SPSS (version 29.0.2.0, IBM, Armonk, NY, USA) and Microsoft Excel (Microsoft, Redmond, WA, USA). This study is exploratory and descriptive in nature, as there are no reliable preliminary studies available in the literature on comparable types of restorations, especially for the crown materials selected in combination with the hybrid abutment crowns.

The aim of this analysis was to identify and describe the differences between the two study groups in terms of implant survival, marginal bone loss, and technical complications. Due to the exploratory nature of this study, formal hypothesis testing was not performed; this is a hypothesis-generating study to prepare for further multicenter studies.

All statistical analyses were performed with a two-sided significance level of α = 0.05. No adjustment was made for multiple comparisons. The primary endpoint of this study was implant loss within the three-year observation period, which was defined as a dichotomous target variable (yes/no). The groups were compared using the chi-square test for independent samples. To quantify the statistical uncertainty, the 95% confidence interval of the difference in the proportion values was calculated.

For the analysis of marginal bone loss, group differences were examined using a two-sided *t*-test for independent samples in the presence of a normal distribution. In the event of deviations from the normal distribution, equivalent non-parametric methods, in particular the Wilcoxon sign rank test, were used.

## 3. Results

### 3.1. Patient Population and Implant Distribution

**Figure 3:**
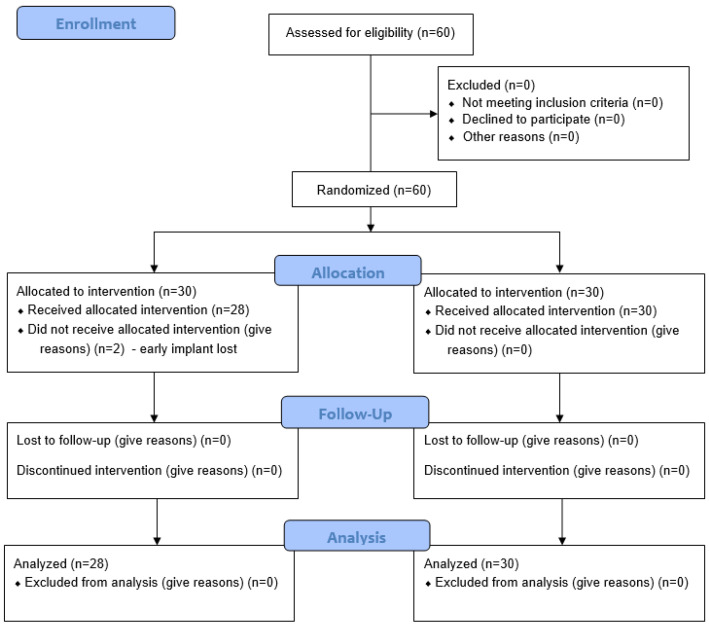
CONSORT flow chart.

Data from a total of 58 patients were included in this evaluation. Two patients were excluded from this study due to early implant loss, within the first 4 weeks after implantation, before prosthetic restoration (Figure 3). No further recruitment was carried out. Due to the subsequent reimplantation, these patients were excluded from further investigation.

The average age was 50.3 years (range: 22–79 years). Of the 58 cases evaluated, 28 patients were in the hybrid ceramic crown group and 30 patients were in the lithium disilicate ceramic group. The total collective consisted of 35 female and 23 male subjects.

A total of 37 implants were inserted in the mandible (63.8%) and 21 implants in the maxilla (36.2%). The distribution of the implant positions is shown in Table 1.

**Figure 4:**
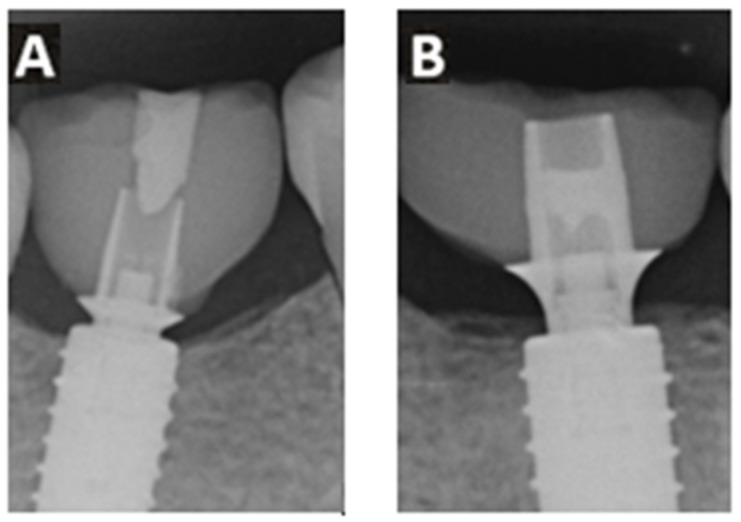
Example X-ray images of the two different Ti-base adhesive bases with gingival height 0.8 mm (**A**) and 2.0 mm (**B**)—after three years.

The implant system used offered two different heights of titanium adhesive bases (Figure 4). In 27 cases, an adhesive base height of 0.8 mm was used, while in the remaining 31 cases, a height of 2.0 mm was selected.

### 3.2. Implant Survival

During the observation period of 36 months after the denture was fitted, no implant loss was recorded, corresponding to a survival rate of 100%.

### 3.3. Changes in Marginal Bone Profile

**Figure 5:**
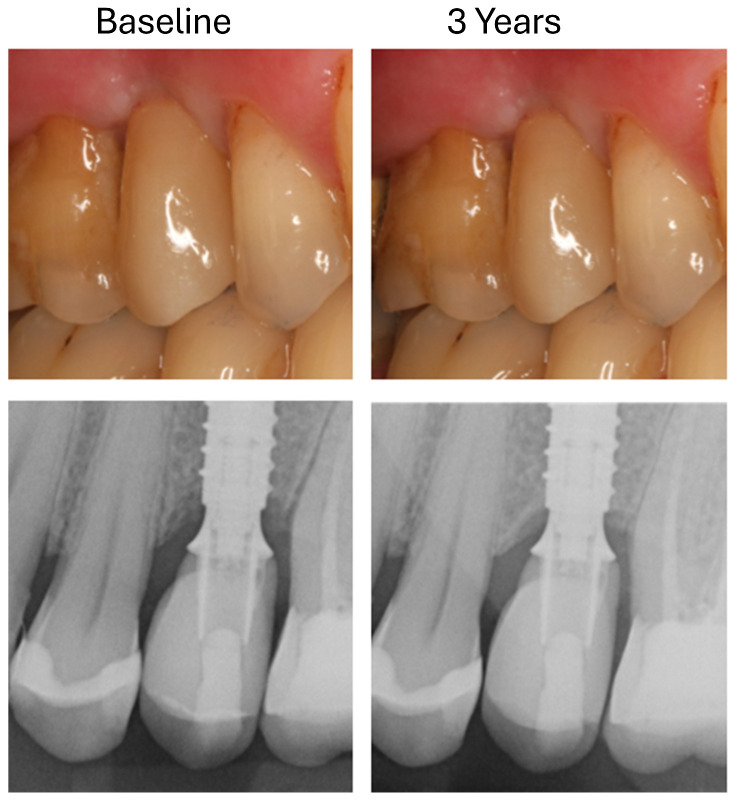
Clinical and radiological documentation of an LS2-hybrid-abutment crown at baseline and after 3 years.

**Figure 6:**
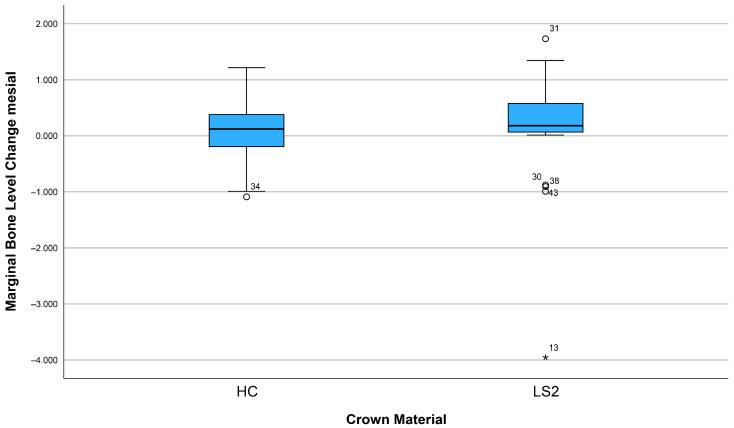
Marginal bone loss depending on the crown material (mesial).

**Figure 7:**
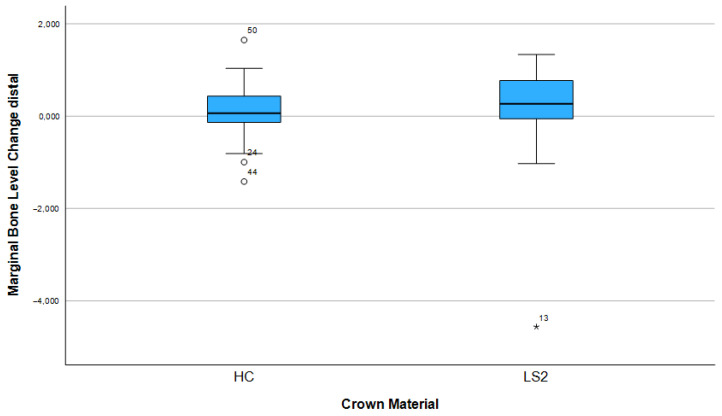
Marginal bone loss depending on the crown material (distal).

After three years, 74.1% (n = 43) of the implants showed bone apposition mesially and 63.8% (n = 37) distally (Figure 5). Marginal bone loss was observed in 25.9% of the implants mesially and 36.2% distally. The mean bone remodeling was 0.13 mm mesially (range: −4.56 to 1.65 mm; SD: 0.88) (Figure 6) and 0.12 mm distally (range: −3.95 to 1.73 mm; SD: 0.78) (Figure 7).

The statistical analysis showed that gender had a significant influence on bone remodeling: in the female patients, a mean bone gain of 0.31 mm (95% CI: 0.12–0.48) was observed mesially and a bone loss of −0.17 mm (95% CI: −0.60–0.27) was observed in the male patients (*p* = 0.024). A similar trend was observed distally, but it was without statistical significance (*p* = 0.674).

Other influencing factors such as crown material (LS2 vs. HC), arch position (maxillary vs. mandibular arch), height of the Ti base (0.8 mm vs. 2.0 mm), implant length, and implant diameter showed no statistically significant influence on bone remodeling, neither mesially nor distally (all *p* > 0.05). The results of the corresponding group comparisons are shown in Table 2.

### 3.4. Technical Complications

Overall, technical complications occurred with both of the materials, with severe damage observed exclusively in the HC group.

**Figure 8:**
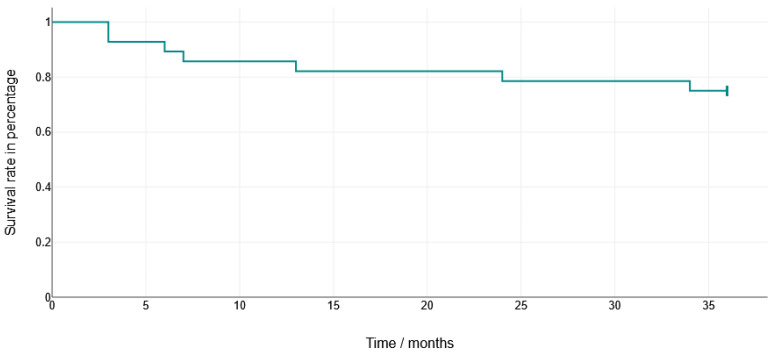
Survival rate of hybrid abutment crowns manufactured using HC. Crowns made from LS2 showed no failures within 36 months.

Crown fractures were observed exclusively in the HC crowns (n = 7; 25.0%), with an average occurrence after 12.86 months. In all of these cases, the crown had to be remade (Figure 8).

Screw loosening occurred in both of the groups: in the HC group, in 14.3% (n = 4; mean time: 17.25 months), and in the LS2 group, in 12.5% (n = 4; mean time: 3.75 months).

Technical complication rates are reported descriptively for the different restorative materials. The Kaplan-Meier analysis of the HC crowns demonstrated a failure-free survival of 71% at 36 months. Because of the small number of events, no statistical comparisons between the materials were carried out.

Loss of bonding (decementation) between the crown and the Ti base was observed exclusively in the HC group in 17.9% (n = 5; mean time: 27.0 months). Rebonding was performed in the dental laboratory.

While minor technical complications such as screw loosening occurred with comparable frequency across the groups, major (decementation) and critical (fracture) complications were observed exclusively in the HC group.

## 4. Discussion

The objective of this clinical study was to evaluate the biological and technical outcomes of hybrid abutment crowns fabricated from either lithium disilicate (LS2) or a high-performance polymer composite (HC), with particular attention to their influence on peri-implant bone remodeling and prosthetic complications. Across the evaluated cohort of 58 patients, no implant loss occurred during the 36-month follow-up period [30], thereby allowing for a valid assessment of the primary endpoint: the influence of the superstructure material on implant survival. The results revealed no significant difference between the two materials in terms of implant survival at both one and three years, aligning with the existing literature that reports similar survival rates regardless of crown material.

This study further demonstrated subtle changes in marginal bone levels around the implants. The average bone remodeling was 0.15 mm mesially and 0.13 mm distally in the LS2 group, and 0.08 mm mesially and 0.12 mm distally in the HC group. These differences were not statistically significant, indicating that both materials are clinically comparable regarding their impact on peri-implant bone stability. These findings are consistent with those of Mangano et al. (2018), who also observed favorable medium-term outcomes with CAD/CAM-fabricated ceramic hybrid abutment crowns [31].

A notable strength of this investigation is its single-center design, which ensured standardized treatment protocols and consistent procedural execution. The influence of operator variability, a well-documented factor in implantology as shown by Jemt et al. (1989) and Chrcanovic et al. (2014), was thus effectively minimized [32,33].

Interestingly, mesial bone remodeling appeared to be gender-dependent: female patients exhibited significantly greater bone apposition than their male counterparts. A similar trend was reported by Cheng et al. (2025) [34], although such gender-specific effects have not been consistently reported across the literature [35].

A limitation of this study is the relatively short observation period of three years and the limited sample size. Although two early implant losses occurred prior to loading, the literature indicates average survival rates ranging from 95% to 98% in the early postoperative years [36]. Within the monitored follow-up period, no further implant failures were recorded.

A limitation of the present investigation is its single-center design, which may reduce the external validity and restrict the generalizability of the findings to other clinical settings. Furthermore, the lack of a formal sample size calculation, due to insufficient prior data for the primary endpoint, could have influenced the statistical reliability of the results. These aspects highlight the need for future multicenter studies with larger sample sizes to confirm and extend the present findings.

Bone apposition was documented in 74.1% of the implants, potentially attributable to the combination of subcrestal implant placement, platform switching, and regenerative surgical protocols, as previously described by Linkevicius et al. (2009) [37]. Only a few cases exhibited marginal bone resorption, with the overall remodeling rates being lower than those commonly reported in similar studies [38].

The employed surgical approach—minimally invasive flap formation with transgingival healing—is considered especially conservative. Reduced tissue trauma and improved healing associated with flapless procedures have been confirmed by Pisoni et al. (2016) [39], although the long-term success appears unaffected by the flap technique itself [40]. The minimal bone loss observed here supports the hypothesis that careful, low-trauma site preparation, possibly aided by single-use drills, contributes to bone preservation.

Postoperative care represents another important factor. According to Atieh et al. (2021), regular peri-implant maintenance significantly reduces marginal bone loss [7]. While this study did not quantify the follow-up care frequency, all of the patients were instructed in proper oral hygiene and selected based on good compliance, suggesting that both home and professional care were adequately provided.

Regarding the prosthetic protocol, a single-stage transgingival healing concept was implemented, minimizing the abutment manipulation. This approach is supported by Vatenas and Linkevičius (2021), Nunes et al. (2025), and Atieh et al. (2017), who reported superior bone maintenance with reduced abutment disconnections [41,42,43].

In summary, no significant material-dependent differences were found regarding marginal bone remodeling, and all of the implants exhibited successful osseointegration.

The adoption of CAD/CAM workflows and the development of novel all-ceramic materials have broadened the indications for aesthetic, metal-free implant restorations. All-ceramic solutions offer excellent biocompatibility and high aesthetic standards [44]. This study focused on comparing the clinical and technical performance of LS2 and HC crowns in single-implant restorations over 36 months.

The hybrid abutment crowns in this study consisted of monolithic CAD/CAM-milled all-ceramic restorations adhesively bonded to prefabricated titanium bases. The occlusal screw channel was sealed with Teflon tape and composite in accordance with clinical standards (Sailer et al., 2018). This type of restoration is well-established in contemporary prosthodontics [45].

A systematic review by Pjetursson et al. (2021) provides survival data for implant-supported all-ceramic single crowns (iSCs): three-year survival rates of 97.6% for veneered and reinforced glass-ceramic iSCs, 97.0% for monolithic glass-ceramics, and 96.3% for veneered zirconia; monolithic zirconia showed a similar performance at 96.1%. Resin nano-ceramics showed a markedly lower survival at 36.3%. The annual complication rate was 3.9% for veneered iSCs and 1.8% for monolithic designs [46].

The limited number of events precluded the robust statistical testing of the factors potentially influencing technical complications. For the variable material, only descriptive analyses were performed, and a Kaplan-Meier curve was provided for the HC crowns. Thus, these results should be interpreted cautiously and considered exploratory, underlining the need for larger studies to evaluate material-related effects.

The findings from the present study are in line with these data. No complications occurred in the LS2 group during the 36-month observation. In contrast, 25% of the HC crowns failed and required replacement. Additionally, decementation events were observed solely in the HC group. This supports the results reported by Zhang et al. (2023), who compared monolithic lithium disilicate and veneered zirconia crowns in an RCT. After three years, the survival rate was 94% in the LS2 group (one fracture) and 100% in the zirconia group [47]. Spitznagel et al. (2021) similarly reported a 100% survival rate over five years for LS2 monolithic restorations on ceramic implants [48].

Our results align with previous studies showing that the elastic modulus affects the peri-implant load transfer. The finite element analyses demonstrated altered stress patterns depending on the material stiffness [19,20,21], while recent work suggested that moduli closer to cortical bone may reduce the biomechanical risks [22]. These findings support the importance of considering an elastic modulus when assessing the implant success.

Pjetursson et al.’s meta-analysis also highlighted the inferior performance of resin-based materials, citing an RCT with 70% survival at five years (n = 25) and a prospective cohort study with 14% survival at one year (n = 50) [46,47,49].

Monolithic ceramics are generally considered more durable than veneered designs. Lower fracture rates and a reduced complication risk favor monolithic crowns, particularly in posterior regions [50,51]. Spitznagel et al. (2017) emphasized the clinical reliability and esthetic benefits of lithium disilicate as a fully anatomical restorative material [52].

## 5. Conclusions

In conclusion, this study affirms the clinical efficacy and predictability of lithium disilicate (LS2) hybrid abutment crowns for posterior single-tooth implant restorations. The three-year outcomes—marked by a 100% survival rate, the absence of complications, and no required reinterventions—support the use of LS2 as a dependable material. In contrast, HC restorations, despite exhibiting a favorable marginal bone stability, were characterized by an increased incidence of technical complications, thereby substantiating previously reported constraints associated with polymer-based materials.

These findings support the clinical recommendation that hybrid abutment crowns—particularly those made from lithium disilicate—offer a reliable treatment modality with stable peri-implant tissue outcomes for at least three years. Nevertheless, further randomized controlled trials with larger cohorts and extended follow-up are needed to validate and expand upon these conclusions.

## Figures and Tables

**Table 1:** Frequencies (n), percentages (%), mean, and standard deviation (SD) values for patient demograaphics in the two groups.

Characteristic	Crown Material		
	Polymer-Infiltrated Hybrid Ceramic (HC)	Lithium Disilicate (LS2)	*p*-Value
Gender			
Male	10 (36%)	13 (43%)	0.553 ns
Female	18 (64%)	17 (57%)	
Age in years	51.8 ± 10.9	48.8 ± 12.9	0.400 ns
Site			
Maxillary arch	10 (36%)	11 (37%)	0.940 ns
Mandibular arch	18 (64%)	19 (63%)	
Premolars Maxillary	6 (21%)	8 (27%)	0.971 ns
Premolars Mandibular	4 (14%)	3 (10%)	
Molars Maxillary	6 (21%)	6 (20%)	
Molars Mandibular	12 (43%)	13 (43%)	

**Table 2:** Factors influencing marginal bone remodeling. * = significant difference between the groups.

Factor	Mesial Bone Remodeling (Mean, 95% CI)	*p*-Value (Mesial)	Distal Bone Remodeling (Mean, 95% CI)	*p*-Value (Distal)
Crown material (LS2)	0.15 (−0.21–0.51)	0.24	0.13 (−0.26–0.53)	0.35
Crown material (HC)	0.08 (−0.12–0.29)		0.12 (−0.13–0.30)	
Gender (female)	0.31 (0.12–0.48)	* 0.024	0.21 (0.02–0.40)	0.674
Gender (male)	−0.17 (−0.60–0.27)		−0.01 (−0.53–0.52)	
Jaw (upper jaw)	0.11 (−0.11–0.32)	0.639	0.11 (−0.18–0.40)	0.622
Jaw (lower jaw)	0.12 (−0.18–0.43)		0.13 (−0.20–0.46)	
Ti–Base height (0.8 mm)	0.04 (−0.37–0.45)	0.64	0.02 (−0.42–0.46)	0.99
Ti–Base height (2.0 mm)	0.18 (0.01–0.36)		0.22 (−0.01–0.44)	
Implant length (9 mm)	0.33 (0.07–0.58)	0.55	0.33 (0.08–0.58)	0.12
Implant length (11 mm)	−0.06 (−0.43–0.32)		0.06 (−0.43–0.32)	
Implant length (13 mm)	0.54 (−0.09–1.18)		0.54 (−0.09–1.18)	
Implant diameter (3.8 mm)	0.04 (−0.20–0.28)	0.619	0.04 (−0.20–0.28)	0.43
Implant diameter (4.4 mm)	−0.24 (−0.07–0.54)		0.24 (−0.07–0.54)	
Implant diameter (5.0 mm)	0.06 (–0.70–0.82)		0.06 (−0.70–0.82)	

## Data Availability

The complete documentation of all the patients enrolled in this study belongs to the authors and is available only upon reasonable request.
